# Supplementary material for: Reconciling Mining with the Conservation of Cave Biodiversity: A Quantitative Baseline to Help Establish Conservation Priorities
Source: PLoS One. 2016 Dec 20;11(12):e0168348. doi: 10.1371/journal.pone.0168348 (PMC5173368; doi:10.1371/journal.pone.0168348)
Supplement: S1 Dataset — (ZIP) [file pone.0168348.s002.zip › Taxa/Serra Norte/SN_2013/N4E/Lista N4E-56.pdf]

CAVIDADE N4E-0056

Classe	Ordem	Fam/Outros	Gên/Outros	Espécie	S-U	U-S	Zon.
Arachnida	Acari	(Parasitengonina)		sp.2		X	E
Arachnida	Acari	Erythraeidae		sp.64	X		E
Arachnida	Acari	Opilioacaridae		sp.1		X	E
Arachnida	Amblypygi	Phrynidae	*Heterophrynus*	*longicornis*		1	E
Arachnida	Araneae	Araneidae		jovem	X	X	E
Arachnida	Araneae	Ctenidae		jovem	2	1	E
Arachnida	Araneae	Filistatidae		sp.1		X	E
Arachnida	Araneae	Mysmenidae	*Microdipoena*	sp.1		X	E
Arachnida	Araneae	Oonopidae		jovem	X	X	E
Arachnida	Araneae	Pholcidae	*Mesabolivar*	sp.1	X	X	E
Arachnida	Araneae	Salticidae		sp.1		X	E
Arachnida	Araneae	Salticidae		sp.2	X		E
Arachnida	Araneae	Scytodidae	*Scytodes*	*eleonorae*	1	1	E
Arachnida	Araneae	Theridiidae	*Theridion*	sp.1	X	X	E
Arachnida	Araneae	Theridiosomatidae		jovem	X	X	E
Arachnida	Opiliones	Stygnidae	*Protimesius*	*laevis*	4		E
Arachnida	Pseudoscorpiones	Chernetidae	*Spelaeochernes*	sp.1		X	E
Arachnida	Schizomida	Hubbardiidae	aff. *Rowlandius*	sp.1		X	E
Chilopoda	Scolopendromorpha	Scolopocryptopidae	*Tidops*	sp.1		1	E
Chilopoda	Scutigeromorpha	Pselliodidae		jovem		1	E
Chilopoda				jovem	1		E
Entognatha	Collembola	Entomobryidae		sp.1	X		E
Entognatha	Collembola	Paronellidae		sp.1		X	E
Insecta	Blattodea	Polyphagidae		sp.1	5	1	E
Insecta	Coleoptera	Carabidae		sp.5	X		E
Insecta	Coleoptera	Scydmaenidae		sp.3	X		E
Insecta	Coleoptera	Staphylinidae	Pselaphinae	sp.22		X	E
Insecta	Diptera	Aulacigastridae		sp.		X	E
Insecta	Diptera	Cecidomyiidae	Cecidomyiinae	sp.		X	E
Insecta	Diptera	Culicidae	*Culex*	sp.	X	X	E
Insecta	Diptera	Dolichopodidae		sp.	X		E
Insecta	Diptera	Psychodidae	Phlebotominae	sp.	X	X	E
Insecta	Diptera	Sciaridae		sp.		X	E
Insecta	Diptera	Tipulidae	Tipulinae	sp.	X		E
Insecta	Heteroptera	Nabidae		sp.	X		E
Insecta	Heteroptera	Reduviidae	Emesinae	sp.		1	E
Insecta	Heteroptera	Reduviidae	*Zelurus*	sp.	6	6	E
Insecta	Hymenoptera	Formicidae	*Gnamptogenys*	sp.1		X	E
Insecta	Hymenoptera	Formicidae	*Pachycondyla*	sp.2	X		E
Insecta	Hymenoptera	Formicidae	*Pheidole*	sp.2	X	X	E
Insecta	Isoptera	Rhinotermitidae	*Heterotermes*	sp.	X		E
Insecta	Isoptera	Termitidae	*Nasutitermes*	sp.	X	X	E
Insecta	Lepidoptera	Noctuoidea		sp.2		1	E
Insecta	Lepidoptera	Noctuoidea		sp.5	1	7	E
Insecta	Lepidoptera	Roeslerstammiidae		sp.		X	E
Insecta	Neuroptera	Myrmeleontidae		jovem	X	X	E
Insecta	Orthoptera	Phalangopsidae	*Paraclodes*	sp.	8	2	E
Insecta	Orthoptera	Phalangopsidae	*Phalangopsis*	jovem	2		E
Insecta	Psocoptera	Psyllipsocidae	*Psyllipsocus*	sp.1		X	E

Insecta	Psocoptera	Ptiloneuridae		jovem		X	E
Insecta	Psocoptera			jovem	X		E
Symphyla		Scutigerellidae		sp.		X	E
Amphibia	Anura	Strabomantidae	*Pristimantis*	cf. *fenestratus*	3		E
Amphibia	Anura			sp.	1		E
Mammalia	Chiroptera	Emballonuridae	*Peropteryx*	*kappleri*	1		E
